# Deep Brain Stimulation in Parkinson's Disease Patients Over 75 Years of Age: A Single‐Institution Retrospective Analysis

**DOI:** 10.1111/cns.70397

**Published:** 2025-05-14

**Authors:** Xin Wang, Jing Wang, Nan Li, Yuqi Wen, Bao Wang, Huijuan Kou, Jian Fu, Hongwen Qu, Chun Qiu, Zixuan Jing, Mingming Su, Zhaohui Zheng, Xuelian Wang, Yan Qu

**Affiliations:** ^1^ Department of Neurosurgery, Tangdu Hospital Air Force Medical University Xi'an Shaanxi China; ^2^ Department of Cardiology, The Second Affiliated Hospital Xi'an Jiaotong University Xi'an Shaanxi China; ^3^ Xi'an Medical University Xi'an Shaanxi China

**Keywords:** advanced age, deep brain stimulation, disease duration, Parkinson's disease, therapeutic effect

## Abstract

**Aim:**

Elderly patients (aged 75 years and older) with Parkinson's disease (PD) are commonly considered unsuitable for deep brain stimulation (DBS) because of its limited benefits for this population and high risk. Understanding therapeutic heterogeneity in terms of effectiveness and safety can help in personalized prognostic and preoperative evaluations.

**Methods:**

The clinical data of 1680 PD patients who underwent DBS surgery in our hospital between April 2007 and July 2023 were retrospectively analyzed. Sixty‐eight of the patients were aged 75 years or older at the time of treatment. The patients were stratified into two groups according to the disease duration: 38 had PD for < 10 years, and 30 had PD for ≥ 10 years. The baseline data, including sex, Hoehn–Yahr scores, PD classification, comorbidities, levodopa responsiveness, anesthesia and operation methods, were compared to confirm the comparability of the results. The Movement Disorder Society‐sponsored revision of the unified Parkinson's disease rating scale‐part 3 (MDS‐UPDRS‐III) score (off‐ or on‐medication condition, off‐medication/on‐stimulation condition, and on‐medication/on‐stimulation condition), levodopa equivalent daily dose (LEDD), 39‐item Parkinson's disease questionnaire (PDQ‐39) score, activities of daily living (ADL) scale score, and adverse events were obtained and compared between the two groups preoperatively and at the postoperative follow‐up.

**Results:**

Among the two groups of patients, no significant differences in sex, PD classification, comorbidities, levodopa responsiveness, anesthesia method, or operation method were observed. In both groups of patients, the postoperative MDS‐UPDRS‐III scores were significantly lower than the preoperative MDS‐UPDRS‐III scores. However, the LEDD, PDQ‐39 score, and ADL score did not decrease significantly in patients with a disease duration ≥ 10 years. The incidence rate of complications was significantly higher in this group of patients.

**Conclusions:**

DBS surgery is effective for improving motor symptoms in elderly PD patients (aged 75 years and older) in the short and long term. Patients with a longer duration of disease (≥ 10 years) cannot easily reduce the use of anti‐Parkinsonian drugs, improve their quality of life, or improve their ability to perform daily living activities. These PD patients often suffer relatively more temporary complications, which should be taken seriously by doctors.

## Graphical abstract

DBS is effective for alleviating motor symptoms in PD patients of advanced age (aged ≥ 75 years). Patients with a longer disease duration (≥ 10 years), regardless of the disease onset time, rarely reduce their use of antiparkinsonian drugs and improve their quality of life, and suffer relatively more short‐term complications.

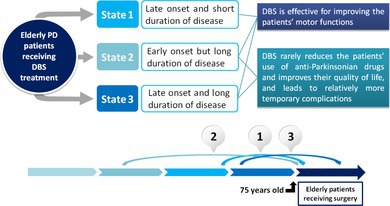

## 1 Introduction

Deep brain stimulation (DBS) is an effective therapy for Parkinson's disease (PD). It has been successfully used to treat PD in select elderly patients and is considered equally effective in this patient group as in young patients owing to long‐term improvements in motor function and a decreased use of medications [1, 2, 3]. However, although no serious adverse events have been observed, elderly patients were reported to have a higher risk of surgery‐related complications [2, 3, 4]. Additionally, the effect of DBS has not been extensively studied in these patients, as they are often excluded from DBS surgery because of concerns of a reduced benefit and more complications [5]. Many neurosurgeons tend to avoid operating on patients aged 65 and older because of their diminished “surgical tolerance”, which is responsible for an increase in the risk of surgical complications [6, 7, 8, 9]. In the consensus and protocol developed by Chinese experts, the age of selected patients is usually limited to 75 years or younger [10, 11]; furthermore, appropriately extending the age range for DBS surgery if a patient is in good physical condition is recommended. Therefore, more careful selection and follow‐up of elderly patients are needed, and more attention should be given to the preoperative assessment and surgical complications [3, 12]. Based on this information, advanced age (age 75 years and older) alone should not be considered a contraindication or exclusion criterion for DBS [4, 6, 13]. Strict risk–benefit assessments should be used to evaluate these elderly patients before surgery.

The clinical outcomes of DBS surgery were compared between elderly patients and younger patients in the above studies. Among elderly PD patients, two main reasons exist for undergoing surgical treatment at an advanced age. One is the late onset of disease; the other is the relatively early onset but long duration of disease, and patients often choose DBS after the “drug honeymoon period”. Understanding the differences in the efficacy and safety of DBS between these two types of patients may be helpful for selecting elderly patients who are more suitable for this surgery, predicting treatment outcomes, assessing surgical risks, performing operations, and optimizing individualized perioperative management.

Therefore, we conducted a retrospective study of patients with idiopathic PD who underwent DBS surgery at Tangdu Hospital between 2007 and 2023. We identified patients who were at least 75 years of age and analyzed the collected data to determine the heterogeneity in elderly PD patients.

## 2 Methods

### 2.1 Participants

Between April 2007 and July 2023, a total of 1680 consecutive patients with idiopathic PD, 68 of whom were aged 75 years or older, underwent DBS surgery at our institution. Sixty‐five patients aged 75 years or older underwent permanent stimulation for more than 1 year. The progression of motor dysfunction in PD patients inevitably leads to severe disability after approximately 10 years of the disease, which can be regarded as an important boundary of motor function, symptom progression, quality of life, and the clinical phase of the patients [14]. Therefore, the patients were classified on a 10‐year basis and divided into two categories according to the disease duration (< 10 years and ≥ 10 years) in this study. The study design and methods were in accordance with the Declaration of Helsinki and approved by the Institutional Review Board of Tangdu Hospital, Air Force Medical University. The ethical approval number was TDLL‐202409‐06.

### 2.2 Clinical Assessments

#### 2.2.1 Baseline Characteristics of the Study Subjects

The age, disease duration, sex, Hoehn–Yahr (H‐Y) stage, PD subclassification, comorbidities, acute levodopa responsiveness, DBS target, and methods of anesthesia and surgery of the patients aged 75 years and older were collected and compared between the two groups.

#### 2.2.2 DBS Surgical Procedures

We used the Leksell (Elekta AB, Stockholm, Sweden) or CRW (Radionics Inc., Burlington, MA, USA) stereotactic systems for DBS surgery. The following models of the implantable electrodes were used: 3389/3387–40 or 40 s (Medtronic Inc., Minneapolis, MN, USA), L301/302 (Beijing Pins Medical Equipment Co. Ltd., Beijing, China), and 1200 (SceneRay Co. Ltd., Suzhou, China).

Some patients underwent 1.5 T or 3.0 T magnetic resonance imaging (MRI) of the brain regions surrounding the targets on the day of the operation. T1‐ or T2‐weighted (T1W or T2W, respectively) imaging (2‐ or 3‐mm slice thickness, 0‐mm slice gap) was selected according to the nucleus. For example, T2W imaging was selected for the subthalamic nucleus (STN), and T1W imaging was selected for the globus pallidus internus (GPi). Stereotactic surgical planning software was used to locate the targets directly and record their coordinates. Bilateral burr holes were often located 0.5–1.0 cm anterior to the coronal suture and 3.5–4.0 cm lateral to the midline. The other patients underwent 1.5 T or 3.0 T MRI scans, including three‐dimensional brain volume (3D BRAVO) and double‐dose contrast‐enhanced imaging, a few days before surgery. On the day of the operation, each patient underwent 1.5 T or 3.0 T MRI scans of the brain regions surrounding the targets after the stereotactic frame was placed. We used the corresponding software to merge the preoperative MR images and MR images obtained after frame placement. After targeting on a sequence of images in which the nucleus was more visible, we planned a trajectory for cannula puncture and electrode placement to avoid the sulci, ventricles, and blood vessels. Finally, two angles on each side of the skull were recorded. All the surgeries were performed by the same surgeon, and all the psychometric scale scores were determined by the same team.

#### 2.2.3 Assessment of the Patient Prognosis After Deep Brain Stimulation Surgery

We compared the Movement Disorder Society‐sponsored revision of the unified Parkinson's disease rating scale‐part 3 (MDS‐UPDRS‐III) scores [these scores in some cases had been recalculated based on the unified Parkinson's disease rating scale‐part 3 (UPDRS‐III) scores and physical examination videos], 39‐item Parkinson's disease questionnaire (PDQ‐39) scores, activities of daily living (ADL) scale scores, and levodopa equivalent daily dose (LEDD) between the two groups before and after surgery to evaluate the effectiveness of DBS surgery. The surgery‐related complications in each group were assessed to evaluate the safety of DBS surgery. Additionally, we used the Mini‐Mental State Examination (MMSE) and Montreal Cognitive Assessment (MoCA) scales to evaluate the cognitive state of PD patients and the Hamilton Anxiety Scale (HAMA) and Hamilton Depression Scale (HAMD) to evaluate their psychological state. Notably, we typically turn on the DBS system for patients 1 month after surgery, and the UPDRS‐III scores at 1 month postsurgery were obtained within 1–3 days after the system was turned on.

### 2.3 Statistical Analysis

Statistical analyses were performed with the Statistical Package for the Social Sciences for Windows (SPSS, version 22.0; IBM, Armonk, NY, USA). Shapiro–Wilk test was used to examine whether the values conformed to a normal distribution. Levene's test was used to assess the homogeneity of variance. The data are presented as the means ± standard deviations (SDs) or medians (the first and third quartiles). Spearman's rank correlation test was used to analyze the correlation between the disease duration and age. Pearson's chi‐squared test, Fisher's exact test, the paired *t* test, the unpaired *t* test, Mann–Whitney *U* test, or the Wilcoxon signed rank test were used for comparisons between two groups or two time points. Data that did not exhibit a normal distribution or have equal variances were analyzed via a nonparametric equivalent. The details of the statistical analyses are presented in each table. For all tests, a *p* value < 0.05 indicated statistical significance.

## 3 Results

### 3.1 Patients' Baseline Characteristics

A total of 68 patients aged 75 years or older who underwent DBS surgery were identified. Thirty‐eight patients had PD for less than 10 years, and 30 patients had PD for 10 years or longer. For all 68 patients, the median age was 80 (78, 83) years, and the median disease duration was 113.5 (81, 157) months. Forty‐seven patients were male, and 21 were female. One patient had H‐Y stage 1.5, 9 had H‐Y stage 2, 10 had H‐Y stage 2.5, 20 had H‐Y stage 3, 25 had H‐Y stage 4, and 3 had H‐Y stage 5. Eight patients had tremor‐dominant (TD) PD, 54 had postural instability and gait disorder (PIGD) PD, and 6 had indeterminate PD. Twelve patients had hypertension, 6 had diabetes, and 6 had heart disease. The mean acute levodopa responsiveness based on the MDS‐UPDRS‐III score was 41.9% ± 24.5%. The leads were placed at the STN (66 patients) or GPi (2 patients) under local (39 patients) or general (29 patients) anesthesia. Only 3 (4.4%) patients underwent unilateral lead placement and stimulation. In addition to the disease duration, the age and H‐Y stage of the patients were significantly different between the two groups (*p* < 0.05). The baseline clinical characteristics of the total patients and those in each group are summarized in Table 1.

**TABLE 1:** Patient demographics and clinical characteristics of the two groups.

Characteristic	Total, *n* = 68	< 10 years, *n* = 38	≥ 10 years, *n* = 30	*p*
Age, years	80 (78, 83)	79.7 ± 2.6	82.9 ± 5.0	0.008^a^
Disease duration, mths	113.5 (81, 157)	87 (68, 104.75)	174 (141.75, 226)	0.000^a^
Sex, *n* (%)				0.889^b^
Male	47 (69.1)	26 (68.4)	21 (70.0)	
Female	21 (30.9)	12 (31.6)	9 (30.0)	
H‐Y stage, *n* (%)				0.020^b^
1.5	1 (1.5)	1 (2.6)	0 (0.0)	
2	9 (13.2)	8 (21.1)	1 (3.3)	
2.5	10 (14.7)	7 (18.4)	3 (10.0)	
3	20 (29.4)	11 (28.9)	9 (30.0)	
4	25 (36.8)	10 (26.3)	15 (50.0)	
5	3 (4.4)	1 (2.6)	2 (6.7)	
Disease subclassification, *n* (%)				0.477^b^
TD	8 (11.8)	6 (15.8)	2 (6.7)	
PIGD	54 (79.4)	29 (76.3)	25 (83.3)	
Indeterminate	6 (8.8)	3 (7.9)	3 (10.0)	
Comorbidities, *n* (%)				
Hypertension	12 (17.6)	7 (18.4)	5 (16.7)	0.851^b^
Diabetes	6 (8.8)	3 (7.9)	3 (10.0)	1.000^c^
Heart disease	6 (8.8)	3 (7.9)	3 (10.0)	1.000^c^
Others	11 (16.2)	5 (13.2)	6 (20.0)	0.518^c^
L‐DOPA response, %	41.9 ± 24.5	39.9 ± 23.9	44.5 ± 25.4	0.444^d^
Lead placement, *n* (%)				0.376^b^
Awake	39 (57.4)	20 (52.6)	19 (63.3)	
Asleep	29 (42.6)	18 (47.4)	11 (36.7)	
DBS targets, *n* (%)				1.000^c^
STN	66 (97.1)	37 (97.4)	29 (96.7)	
GPi	2 (2.9)	1 (2.6)	1 (3.3)	
Unilateral stimulation, *n* (%)	3 (4.4)	1 (2.6)	2 (6.7)	0.579^c^

*Note:* The data are presented as the numbers of patients (%), means ± SDs, or medians (Q1, Q3), unless indicated otherwise. *p* values are for comparisons of the two groups.

Abbreviations: DBS, deep brain stimulation; GPi, globus pallidus internus; H‐Y stage, Hoehn and Yahr stage; L‐DOPA response, UPDRS III acute levodopa responsiveness; PIGD, postural instability and gait disorder; Q1, quartile one; Q3, quartile three; SD, standard deviation; STN, subthalamic nucleus; TD, tremor‐dominant.

^a^ Mann–Whitney *U* test was used.

^b^ Pearson's chi‐squared test was used.

^c^ Fisher's exact test was used.

^d^ The unpaired *t* test was used.

H‐Y stages 1.5, 2, and 2.5 and H‐Y stages 4 and 5 were combined into one category for Pearson's chi‐squared test. TD and indeterminate statuses were combined into one category for Pearson's chi‐squared test.

### 3.2 Correlation Between the Disease Duration and Age

**FIGURE 1:**
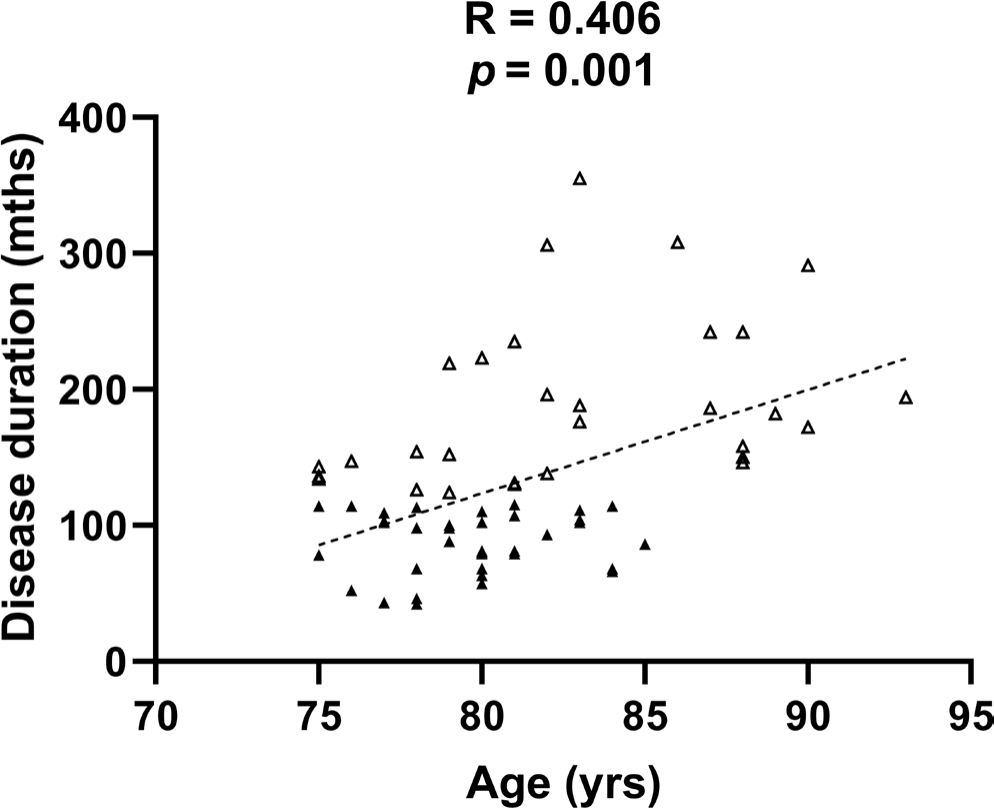
Correlation between the disease duration and age. Scatter plots showing the correlation between the disease duration and age in PD patients (≥ 75 years old) receiving DBS surgery. The filled triangles and outlined triangle represent the groups with disease durations < 120 months and ≥ 120 months, respectively. The disease duration was positively correlated with age (*R* = 0.406, *p* = 0.0006, two‐tailed, *n* = 38/30, number of patients in the two groups). Spearman's rank correlation test was used to analyse the correlation between the disease duration and age.

After the correlation analysis, we found that the disease duration was positively correlated with age in our elderly PD patients (*R* = 0.406, *p* = 0.0006). Figure 1 shows the correlation between the disease duration and age in the total patients and in each group.

### 3.3 MDS‐UPDRS‐III Motor Scores

The pre‐DBS MDS‐UPDRS‐III scores were significantly different in the OFF medication state [37.1 ± 12.2 in the disease duration (DD) < 10 years group and 44.6 ± 12.9 in the DD ≥ 10 years group, *p* = 0.016] and were not significantly different in the ON medication state (22.9 ± 12.1 in the DD < 10 years group and 24.9 ± 11.5 in the DD ≥ 10 years group, *p* = 0.494) between the two groups. One month after DBS surgery, the MDS‐UPDRS‐III scores for OFF medication/ON stimulation (22.7 ± 9.9 in the DD < 10 years group and 24.8 ± 10.2 in the DD ≥ 10 years group, *p* = 0.383) and ON medication/ON stimulation (17.8 ± 9.7 in the DD < 10 years group and 19.3 ± 9.6 in the DD ≥ 10 years group, *p* = 0.539) were not significantly different between the two groups. Twelve months after DBS surgery, the MDS‐UPDRS‐III scores were not significantly different for OFF medication/ON stimulation (24.1 ± 10.1 in the DD < 10 years group and 27.9 ± 11.5 in the DD ≥ 10 years group, *p* = 0.165) but were significantly different for ON medication/ON stimulation (18.7 ± 9.4 in the DD < 10 years group and 24.0 ± 10.6 in the DD ≥ 10 years group, *p* = 0.038) between the two groups. The changes from ON medication scores at baseline to OFF medication/ON stimulation scores at 12 months after surgery [2.3 (−16 to 13) in the DD < 10 years group and 3.2 (−20 to 18) in the DD ≥ 10 years group, *p* = 0.698] were not significantly different between the two groups. The changes from ON medication scores at baseline to ON medication/ON stimulation scores at 12 months after surgery [−3.1 (−15 to 3) in the DD < 10 years group and − 0.7 (−21 to 11) in the DD ≥ 10 years group, *p* = 0.008] were significantly different between the two groups.

In the DD < 10 years group, the changes in the MDS‐UPDRS‐III scores from OFF medication at baseline to OFF medication/ON stimulation at 1 and 12 months after DBS surgery were statistically significant (*p* = 0.000). The changes in scores from ON medication at baseline to ON medication/ON stimulation at 1 and 12 months after DBS surgery were also statistically significant (*p* = 0.000). In the DD ≥ 10 years group, the changes in the MDS‐UPDRS‐III scores from OFF medication at baseline to OFF medication/ON stimulation at 1 and 12 months after DBS surgery were statistically significant (*p* = 0.000). The changes in scores from ON medication at baseline to ON medication/ON stimulation 1 month after DBS surgery were also statistically significant (*p* = 0.000). However, the changes in scores from ON medication at baseline to ON medication/ON stimulation 12 months after DBS surgery were not statistically significant (*p* = 0.624).

Table 2 shows the pre‐DBS and post‐DBS MDS‐UPDRS‐III scores of the two groups.

**TABLE 2:** Baseline and postsurgical MDS‐UPDRS‐III motor scores of the two groups.

		Disease duration < 10 years (*n* = 38)						Disease duration ≥ 10 years (*n* = 30)						*p* ^b^	*p* ^c^
		OFF med	ON med	OFF med/ON stim	*p*	ON med/ON stim	*p*	OFF med	ON med	OFF med/ON stim	*p*	ON med/ON stim	*p*		
Pre‐DBS scores		37.1 ± 12.2	22.9 ± 12.1					44.6 ± 12.9	24.9 ± 11.5					0.016^e^	0.494^e^
Post‐DBS scores	1 month			22.7 ± 9.9	0.000^d^	17.8 ± 9.7	0.000^d^			24.8 ± 10.2	0.000^d^	19.3 ± 9.6	0.000^d^	0.383^e^	0.539^e^
	12 months			24.1 ± 10.1 (*n* = 35)	0.000^d^	18.7 ± 9.4 (*n* = 35)	0.000^d^			27.9 ± 11.5 (*n* = 28)	0.000^d^	24.0 ± 10.6 (*n* = 28)	0.624^d^	0.165^e^	0.038^e^
Changes^a^				2.3 (−16 to 13)		−3.1 (−15 to 3)				3.2 (−20 to 18)		−0.7 (−21 to 11)		0.698^f^	0.008^f^

*Note:* The data are presented as the means ± SDs or means (minima to maxima). *p* values are for comparisons of OFF medication (ON medication) scores at baseline and OFF medication/ON stimulation (ON medication/ON stimulation) scores at one and 12 months postoperatively.

Abbreviations: DBS, deep brain stimulation; MDS‐UPDRS‐III, Movement Disorder Society‐sponsored revision of the unified Parkinson's disease rating scale‐part 3; SD, standard deviation.

^a^ Changes between ON medication scores at baseline and OFF medication/ON stimulation scores or ON medication/ON stimulation scores at 12 months postoperatively.

^b^ *p* values are for comparisons of OFF medication scores at baseline, OFF medication/ON stimulation scores postoperatively, or the changes in scores between the two groups.

^c^ *p* values are for comparisons of ON medication scores at baseline, ON medication/ON stimulation scores postoperatively, or the changes in scores between the two groups.

^d^ The paired *t* test was used.

^e^ The unpaired *t* test was used.

^f^ Mann–Whitney *U* test was used.

### 3.4 Levodopa Equivalent Daily Dose

No statistically significant difference in the LEDD at baseline was observed between the two groups (650.6 ± 352.9 in the DD < 10 years group and 680.1 ± 286.6 in the DD ≥ 10 years group, *p* = 0.712). From baseline to 12 months after DBS surgery, the LEDD was significantly decreased in the DD < 10 years group (650.6 ± 352.9 at baseline, 523.6 ± 275.3 at 12 months after surgery, *p* = 0.005). However, it did not decrease significantly in the DD ≥ 10 years group (680.1 ± 286.6 at baseline, 659.8 ± 237.3 at 12 months after surgery, *p* = 0.545). The changes in the LEDD from baseline to 12 months after DBS surgery were not significantly different between the two groups (−85.6 ± 167.3 in the DD < 10 years group and − 27.8 ± 240.0 in the DD ≥ 10 years group, *p* = 0.547).

### 3.5 Quality of Life and Activities of Daily Living Scale Scores

A statistically significant difference in the PDQ‐39 scores at baseline was not observed between the two groups (42.5 ± 17.2 in the DD < 10 years group and 47.1 ± 17.1 in the DD ≥ 10 years group, *p* = 0.272). From baseline to 12 months after DBS surgery, the PDQ‐39 scores decreased significantly in the DD < 10 years group (42.5 ± 17.2 at baseline, 38.0 ± 17.5 at 12 months after surgery, *p* = 0.010). Significant decreases were not observed in the DD ≥ 10 years group (47.1 ± 17.1 at baseline, 45.8 ± 19.2 at 12 months after surgery, *p* = 0.197). The changes in these scores from baseline to 12 months after DBS surgery were not significantly different between the two groups (−3.8 ± 8.2 in the DD < 10 years group and − 1.9 ± 7.4 in the DD ≥ 10 years group, *p* = 0.340).

No statistically significant difference in the ADL scores at baseline was observed between the two groups [20 (18, 23) in the DD < 10 years group and 21.5 (18, 28.5) in the DD ≥ 10 years group, *p* = 0.207]. From baseline to 12 months after DBS surgery, the ADL scores decreased significantly in the DD < 10 years group [20 (18, 23) at baseline, 20 (17, 23) at 12 months after surgery, *p* = 0.005]. No significant decreases were observed in the DD ≥ 10 years group [21.5 (18, 28.5) at baseline, 20 (18, 30) at 12 months after surgery, *p* = 0.859]. The changes in these scores from baseline to 12 months after DBS surgery were not significantly different between the two groups (−0.9 ± 1.9 in the DD < 10 years group and − 0.1 ± 2.1 in the DD ≥ 10 years group, *p* = 0.098).

### 3.6 Cognitive Measures and Psychological Scales

We chose the MMSE and MoCA scales to evaluate the cognitive state of patients. The MMSE scores were significantly lower in the DD ≥ 10 years group than in the DD < 10 years group at baseline [26 (24, 28) in the DD < 10 years group and 24 (22.75, 26) in the DD ≥ 10 years group, *p* = 0.006]. From baseline to 12 months after DBS surgery, the MMSE scores decreased significantly in the DD ≥ 10 years group [24 (22.75, 26) at baseline, 24 (21.25, 25) at 12 months after surgery, *p* = 0.024]. However, no significant decreases were detected in the DD < 10 years group [26 (24, 28) at baseline, 26 (24, 27) at 12 months after surgery, *p* = 0.091]. The changes in these scores from baseline to 12 months after DBS surgery were not significantly different between the two groups [0 (−1, 1) in the DD < 10 years group and − 1 (−2, 1) in the DD ≥ 10 years group, *p* = 0.396].

The MoCA scores were significantly lower in the DD ≥ 10 years group than in the DD < 10 years group at baseline (25.4 ± 2.8 in the DD < 10 years group and 23.5 ± 3.1 in the DD ≥ 10 years group, *p* = 0.014). From baseline to 12 months after DBS surgery, the MoCA scores were significantly decreased both in the DD < 10 years group (25.4 ± 2.8 at baseline, 24.7 ± 3.0 at 12 months after surgery, *p* = 0.015) and in the DD ≥ 10 years group (23.5 ± 3.1 at baseline, 22.6 ± 3.2 at 12 months after surgery, *p* = 0.003). The changes in these scores from baseline to 12 months after DBS surgery were not significantly different between the two groups (−0.7 ± 1.5 in the DD < 10 years group and − 1.0 ± 1.6 in the DD ≥ 10 years group, *p* = 0.435).

Additionally, we chose the HAMA and HAMD scales to evaluate the psychological state of the patients. No statistically significant differences in the HAMA scores at baseline were observed between the two groups [4 (3, 5) in the DD < 10 years group and 4 (3, 6) in the DD ≥ 10 years group, *p* = 0.298]. From baseline to 12 months after DBS surgery, the HAMA scores did not increase significantly in either the DD < 10 years group [4 (3, 5) at baseline, 4 (3, 5) at 12 months after surgery, *p* = 0.110] or the DD ≥ 10 years group [4 (3, 6) at baseline, 5 (3.25, 6) at 12 months after surgery, *p* = 0.145]. The changes in these scores from baseline to 12 months after DBS surgery were not significantly different between the two groups [0 (0, 1) in the DD < 10 years group and 0 (0, 1) in the DD ≥ 10 years group, *p* = 0.902].

Statistically significant differences in the HAMD scores at baseline were not observed between the two groups [5 (4, 6) in the DD < 10 years group and 5 (4, 7) in the DD ≥ 10 years group, *p* = 0.855]. From baseline to 12 months after DBS surgery, the HAMD scores increased significantly in the DD ≥ 10 years group [5 (4, 7) at baseline, 5 (4.25, 7) at 12 months after surgery, *p* = 0.038]. No significant increases were detected in the DD < 10 years group [5 (4, 6) at baseline, 5 (5, 7) at 12 months after surgery, *p* = 0.096]. The changes in these scores from baseline to 12 months after DBS surgery were not significantly different between the two groups (0.4 ± 1.4 in the DD < 10 years group and 0.5 ± 1.2 in the DD ≥ 10 years group, *p* = 0.769).

Table 3 shows the medication and scale score changes in the two groups before and 12 months after DBS surgery.

**TABLE 3:** Medication and scale score changes before and 12 months after DBS in the two groups.

Parameter	Disease duration < 10 years				Disease duration ≥ 10 years				*p* ^a^	*p* ^b^
	Pre‐DBS (*n* = 38)	Post‐DBS (*n* = 35)	*p*	Changes	Pre‐DBS (*n* = 30)	Post‐DBS (*n* = 28)	*p*	Changes		
LEDD, mg	650.6 ± 352.9	523.6 ± 275.3	0.005^c^	−85.6 ± 167.3	680.1 ± 286.6	659.8 ± 237.3	0.545^c^	−27.8 ± 240.0	0.712^e^	0.547^f^
PDQ‐39	42.5 ± 17.2	38.0 ± 17.5	0.010^c^	−3.8 ± 8.2	47.1 ± 17.1	45.8 ± 19.2	0.197^c^	−1.9 ± 7.4	0.272^e^	0.340^e^
ADL	20 (18, 23)	20 (17, 23)	0.005^d^	−0.9 ± 1.9	21.5 (18, 28.5)	20 (18, 30)	0.859^d^	−0.1 ± 2.1	0.207^f^	0.098^e^
MMSE	26 (24, 28)	26 (24, 27)	0.091^d^	0 (−1, 1)	24 (22.75, 26)	24 (21.25, 25)	0.024^c^	−1 (−2, 1)	0.006^f^	0.396^f^
MoCA	25.4 ± 2.8	24.7 ± 3.0	0.015^c^	−0.7 ± 1.5	23.5 ± 3.1	22.6 ± 3.2	0.003^c^	−1.0 ± 1.6	0.014^e^	0.435^e^
HAMA	4 (3, 5)	4 (3, 5)	0.110^d^	0 (0, 1)	4 (3, 6)	5 (3.25, 6)	0.145^d^	0 (0, 1)	0.298^f^	0.902^f^
HAMD	5 (4, 6)	5 (5, 7)	0.096^d^	0.4 ± 1.4	5 (4, 7)	5 (4.25, 7)	0.038^d^	0.5 ± 1.2	0.855^f^	0.769^e^

*Note:* The data are presented as the means ± SDs or medians (Q1, Q3).

Abbreviations: ADL, activities of daily living scale; DBS, deep brain stimulation; HAMA, Hamilton Anxiety Scale; HAMD, Hamilton Depression Scale; LEDD, levodopa equivalent daily dose; MMSE, Mini‐Mental State Examination; MoCA, Montreal Cognitive Assessment; PDQ‐39, 39‐item Parkinson's disease questionnaire; Q1, quartile one; Q3, quartile three; SD, standard deviation.

^a^ *p* values are for comparisons of the two groups at baseline.

^b^ *p* values are for comparisons of the changes between the two groups.

^c^ The paired *t* test was used.

^d^ The Wilcoxon signed rank test was used.

^e^ The unpaired *t* test was used.

^f^ Mann–Whitney *U* test was used.

### 3.7 Results of the Assessments of the Population Aged > 85 Years

The MDS‐UPDRS‐III motor scores and the medication and scale score changes before and 1 or 12 months after DBS surgery were individually evaluated in the population above 85 years of age and are presented in Table 4 and Table 5, respectively. In these two tables, the change in the MDS‐UPDRS‐III scores from OFF medication at baseline (44.5 ± 12.8) to OFF medication/ON stimulation 1 month after DBS surgery (23.1 ± 10.4) was statistically significant (*p* = 0.000). The change in scores from OFF medication at baseline (44.5 ± 12.8) to OFF medication/ON stimulation 12 months after DBS surgery (25.1 ± 12.0) was statistically significant (*p* = 0.000). The change in scores from ON medication at baseline (25.9 ± 12.6) to ON medication/ON stimulation 1 month after DBS surgery (18.1 ± 9.0) was also statistically significant (*p* = 0.008). The MoCA scores decreased significantly (*p* = 0.034) from baseline (23.7 ± 2.8) to 12 months after DBS surgery (22.1 ± 2.6). The HAMD scores increased significantly (*p* = 0.014) from baseline [5 (3, 6)] to 12 months after DBS surgery [5 (4, 7)]. No statistically significant differences in the other comparisons were observed (*p* > 0.05).

**TABLE 4:** Baseline and postsurgical MDS‐UPDRS‐III motor scores of the population aged > 85 years (*n* = 11).

		OFF med	ON med	OFF med/ON stim	*p* ^a^	ON med/ON stim	*p* ^b^
Pre‐DBS scores		44.5 ± 12.8	25.9 ± 12.6				
Post‐DBS scores	1 month			23.1 ± 10.4	0.000	18.1 ± 9.0	0.008
	12 months			25.1 ± 12.0 (*n* = 10)	0.000	21.2 ± 10.4 (*n* = 10)	0.299
Changes^c^				0.6 ± 11.4		−3.3 ± 9.5	

*Note:* The data are presented as the means ± SDs.

Abbreviations: DBS, deep brain stimulation; MDS‐UPDRS‐III, Movement Disorder Society‐sponsored revision of the unified Parkinson's disease rating scale‐part 3; SD, standard deviation.

^a^ *p* values are for comparisons of OFF medication scores at baseline and OFF medication/ON stimulation scores at one and 12 months postoperatively.

^b^ *p* values are for comparisons of ON medication scores at baseline and ON medication/ON stimulation scores at one and 12 months postoperatively.

^c^ Changes between ON medication scores at baseline and OFF medication/ON stimulation scores or the ON medication/ON stimulation scores at 12 months postoperatively.

A paired *t* test was used.

**TABLE 5:** Medication and scale score changes before and 12 months after DBS in the population aged > 85 years (*n* = 11).

	Pre‐DBS (*n* = 11)	Post‐DBS (*n* = 10)	*p*	Changes
LEDD, mg	624.0 ± 273.7	636.4 ± 210.0	0.710^a^	25.0 ± 206.1
PDQ‐39	40.2 ± 14.7	39.3 ± 16.3	0.204^a^	−3.6 ± 8.3
ADL	20 (19, 25)	19 (17, 24)	0.079^b^	−1.0 ± 1.6
MMSE	24.1 ± 2.4	22.9 ± 1.9	0.147^a^	−0.9 ± 1.8
MoCA	23.7 ± 2.8	22.1 ± 2.6	0.034^a^	−1.5 ± 1.9
HAMA	4.8 ± 1.9	5.0 ± 1.9	0.792^a^	0.1 ± 1.2
HAMD	5 (3, 6)	5 (4, 7)	0.014^b^	1 (0, 1.25)

*Note:* The data are presented as the means ± SDs or medians (Q1, Q3).

Abbreviations: ADL, activities of daily living scale; DBS, deep brain stimulation; HAMA, Hamilton Anxiety Scale; HAMD, Hamilton Depression Scale; LEDD, levodopa equivalent daily dose; MMSE, Mini‐Mental State Examination; MoCA, Montreal Cognitive Assessment; PDQ‐39, 39‐item Parkinson's disease questionnaire; Q1, quartile one; Q3, quartile three; SD, standard deviation.

^a^ The paired *t* test was used.

^b^ The Wilcoxon signed rank test was used.

### 3.8 Adverse Events

The adverse events were classified into surgical and medical complications. The two most common complications related to surgery were transient psychiatric deterioration and cognitive impairment, the incidences of which were not significantly different between the two groups (psychiatric deterioration: 8 patients in the DD < 10 years group, 12 patients in the DD ≥ 10 years group, *p* = 0.089; cognitive impairment: 7 patients in the DD < 10 years group, 7 patients in the DD ≥ 10 years group, *p* = 0.619). The incidence of surgical complications was not significantly different between the two groups (13 patients in the DD < 10 years group and 16 patients in the DD ≥ 10 years group, *p* = 0.113). Additionally, the incidences of a prolonged hospital stay and medical complications were not significantly different between the two groups (9 patients in the DD < 10 years group and 11 patients in the DD ≥ 10 years group, *p* = 0.243). However, the incidence of total adverse events was significantly higher in the DD ≥ 10 years group than in the DD < 10 years group (16 patients in the DD < 10 years group and 21 patients in the DD ≥ 10 years group, *p* = 0.022). Table 6 shows the postoperative complications and adverse effects experienced by the two groups.

**TABLE 6:** Complications and adverse effects observed in the two groups.

	Total (*n* = 68)	Disease duration < 10 years (*n* = 38)	Disease duration ≥ 10 years (*n* = 30)	*p*
Surgical	29	13 (34.2%)	16 (53.3%)	0.113
Transient psychiatric deterioration^a^	20	8 (21.1%)	12 (40.0%)	0.089
Transient cognitive impairment	14	7 (18.4%)	7 (23.3%)	0.619
Transient consciousness disorder	2	1	1	
Intracranial hemorrhage	2	1	1	
Edema around the lead	3	1	2	
Seizure	1	1	0	
Worsening of gait	1	0	1	
Worsening of speech	1	1	0	
Poor wound healing	1	0	1	
Wound infection	1	1	0	
System removal	1	1	0	
Lead migration	0	0	0	
Medical	20	9 (23.7%)	11 (36.7%)	0.243
Pneumonia	5	2	3	
Urinary infection	1	0	1	
Pulmonary embolism	0	0	0	
Prolonged hospital stay^b^	20	9 (23.7%)	11 (36.7%)	0.243
Readmission within 3 months of discharge	3	1	2	
Death within 1 year	1	0	1	
Total (patients)	37	16 (42.1%)	21 (70.0%)	0.022

^a^ Mainly referring to transient delirium and confusion.

^b^ Referring to more than 10 days of postoperative hospitalization.

Pearson's chi‐squared test was used.

## 4 Discussion

Compared with young patients with PD, patients with PD of an advanced age have their own characteristics. They are more likely to experience axial symptoms, such as gait and postural impairment presenting with recurrent falls [15]; psychotic symptoms [16, 17], such as visual hallucinations; and dementia and dysfunction of other brain functions due to brain atrophy [18], which means greater neuronal loss. These patients have a poorer response to levodopa [19, 20], and resting tremors are observed more frequently as their initial symptom [15, 16, 21]. These clinical features may lead to some differences in their postoperative outcomes compared with those of younger patients.

Advanced‐aged patients (aged ≥ 75 years) are commonly considered unsuitable for DBS surgery because of its limited benefits for this group and high risk, or they are recruited only after strict screening and evaluation. The findings concerning efficacy and complications between elderly and young patients were inconsistent in previous comparative studies. Our center has dedicated extensive efforts to studying the effectiveness and safety of DBS surgery for PD patients of advanced age. We found that short‐term (1 month) DBS was effective at alleviating motor symptoms in elderly (≥ 75 years old) PD patients, regardless of their disease duration. Patients with a longer duration of disease (≥ 10 years), who often have more severe symptoms and are of a more advanced age, have difficulty reducing their use of anti‐PD drugs after DBS surgery, and improvements in quality of life and activities of daily living are not significant, indicating that their surgical benefits are limited. These patients had poorer postoperative cognitive and depressive statuses, and the probability of complications after DBS surgery was higher.

In our baseline research, we found that age and the H‐Y stage were higher in the group with a longer duration of disease (≥ 10 years). Understandably, the H‐Y stage, which itself represents the severity of PD, is related to the disease duration. A longer disease duration inevitably indicates more severe symptoms of the disease. Moreover, we found that age was positively correlated with the disease duration in these patients after further analysis, which might be an epidemiological feature of elderly patients with chronic diseases. Therefore, we believe that the data from the two groups were still comparable, but we must realize that more very elderly patients had been included in the group with a longer duration of disease when any conclusions were drawn.

In terms of MDS‐UPDRS‐III motor scores, Derost et al. reported no significant difference between young and old patients in terms of the acute effects of STN‐DBS [19]. Vesper et al. reported that the changes in the UPDRS‐III motor scores were not significantly different between the two groups (aged < 65 years and aged ≥ 65 years) [3]. In our study, the motor scores were significantly different in the OFF medication state between the two groups, revealing that patients with longer durations of disease had more severe motor symptoms in this state and that the motor function of PD patients gradually declines as the disease progresses. Postoperative motor symptoms were significantly alleviated in both groups in the short and long term, except for those in the longer duration of disease group on ON medication/ON stimulation at 12 months after DBS surgery. These results indicated that DBS had limited additional long‐term effects on PD patients with longer durations of disease when they were in a state of ON medication. One important reason for this result may be that for patients with severe symptoms and a very low quality of life, a reduction in the dosage of medication after surgery is difficult, resulting in a greater weight of the therapeutic effect of medication on their comprehensive treatments. STN‐DBS and levodopa independently decrease motor symptom severity in PD patients to a similar magnitude, but their combined effect is greater than that of either treatment alone, suggesting therapeutic synergism [22]. Our data obtained at 12 months postsurgery indicated that, compared with preoperative medication therapy alone, the synergistic effect of stimulation diminished, although DBS therapy alone was still effective. However, compared with stimulation alone at the same time point, the synergistic effect of medication might still be present. Considering our subsequent findings regarding LEDD and the symptomatic fluctuations and side effects due to the medication, we can consider reducing the dosage for patients with longer durations of disease after long‐term DBS in the future, especially for those who have shown good response to stimulation or have experienced severe side effects from medication. Unlike Vesper's study, which compared patients classified by age, we found a significant difference in the change in MDS‐UPDRS‐III scores before and after surgery between the two populations of elderly patients grouped by disease duration, suggesting that classifying patients based on their disease duration could yield more accurate results. Additionally, Du T et al. reported that age and disease duration played unique roles in controlling the symptoms of PD patients (mean age of 62.47 ± 7.73 years) treated with STN‐DBS. Patients with a long disease duration and early disease onset benefit from an increase in therapeutic efficacy in treating general, rigid, and axial symptoms after a long period [23].

Wu W et al. reported that the Barthel index for activities of daily living (ADL‐Barthel) score and LEDD were significantly improved in elderly patients (≥ 75 years) with PD at the 2‐year follow‐up [24]. In our study, in contrast to the substantial reductions in the LEDD, PDQ‐39, and ADL scale scores observed in the group with a disease duration < 10 years, these values did not decrease significantly in the group with a longer disease duration, indicating that the benefits of DBS in reducing the dosage of medication, improving quality of life, and the ability to perform activities of daily living in this group were relatively small. These results might be because this population of patients had a more severe impairment of motor function and suffered more nonmotor manifestations, which affected their quality of life and activities of daily living more seriously. Consequently, from this perspective, these patients might require more use of anti‐PD medications and more optimized drug combination therapy in addition to an adjustment of DBS parameters. Derost et al. reported that the quality of life assessed with the PDQ‐39 improved in subscales evaluating mobility, activities of daily living, emotion and stigma, cognition, and communication in young patients (< 65 years old) compared with those in older patients (≥ 65 years old) [19]. Vesper et al. reported significant differences in overall performance, as determined by ADL scale scores, whereas changes in the use of levodopa medication were not significantly different between the younger and elderly patient groups [3]. Russmann et al. observed that patients over 70 years of age had worse UPDRS‐III motor scores when on medication, despite the little reduction in medication usage, and that their activities of daily living also worsened after the operation [20]. These results collectively revealed that a poor medication response and minimal improvements in quality of life and the ability to perform activities of daily living were common characteristics of elderly PD patients who underwent DBS surgery. Therefore, for elderly patients with advanced PD who have a prolonged disease duration, the expected treatment outcomes should be explained to them and their family members before surgery. While DBS can improve patients' motor symptoms, it may not significantly enhance their quality of life or reduce the medication dosage. Patients will require more rehabilitation and nursing care to alleviate the rapid progression of the disease and its complications.

In this study, the MMSE and MoCA scores were lower at the 12‐month postoperative follow‐up in both groups, although the magnitude of the changes appeared to be relatively small compared with the baseline scores. Transient cognitive impairment, as a main complication, was found in a significant number of our patients. DBS is an effective treatment for PD patients but can be complicated by side effects such as cognitive impairment [25]. Smeding HM et al. reported that PD patients experienced cognitive decline after STN‐DBS surgery [26]. Del Bene VA et al. showed that bilateral STN‐DBS and unilateral left stimulation worsened verbal fluency performance, and regardless of the stimulated hemisphere, delayed recall declined modestly over time versus the baseline measurement [27]. Additionally, the lead trajectory passing through the caudate nucleus for STN‐DBS may affect PD patients' cognitive flexibility [28]. Cognitive changes following the DBS intervention are heterogeneous and related to disease progression, medication side effects, and microlesional effects [29]. In addition to surgery and electrical stimulation, advanced age and PD itself can also affect patients' cognitive function. Age is associated with worse outcomes for most of the measures examined and has similar negative effects on episodic memory and executive functions [30]. Increased age is associated with normative declines in cognitive functioning [31]. PD patients typically exhibit some degree of cognitive dysfunction [29]. Moreover, PD is one of the most common age‐related brain disorders. Compared with age‐matched groups without PD, people with PD exhibit a more rapid decline in numerous cognitive domains, particularly the executive, attentional, and visuospatial domains, as well as memory. The majority of PD patients will develop dementia if they survive for more than 10 years after the diagnosis [32]. In this study, a follow‐up period of 12 months was sufficient to exclude the effects of the lead placement surgical procedure. However, we were still unable to determine whether this difference in the cognitive assessment scale scores between the baseline and follow‐up resulted from continuous bilateral STN stimulation or progressive cognitive decline in elderly patients with PD. This analysis requires the acquisition of data 2 to 3 months after surgery, which is a sufficiently short period to avoid cognitive decline due to disease progression or aging. Similarly, the difference in preoperative cognitive levels between the two groups was also related to differences in age and disease severity.

The HAMD scores increased after DBS surgery in the group with a longer disease duration. This result may be related to the greater impairments in motor function and reduced quality of life in these patients. They may feel disheartened as the surgery did not significantly improve their lives or reduce their medication dosage. Unlike the findings of this study, the results of a meta‐analysis indicated a slight improvement in anxiety and depression after STN‐DBS [33, 34]. Moreover, these DBS effects are weakly moderated by age, the levodopa dose at follow‐up, and the stimulation duration [34]. Our HAMD score results suggest that the reasons for the aggravation of depression in superelderly PD patients after surgery are more complex and may be due to their unchanged quality of life, severe disease progression, and mild cognitive decline.

Another reason that elderly PD patients undergo DBS surgery later is that they have both a late onset and a long duration of disease. These patients were usually over 85 years old and comprised only a comparatively small proportion. In this study, the group of patients with a disease duration ≥ 10 years included all patients aged > 85 years. Thus, the group of patients with a longer duration of disease tended to be older and of a more advanced age. This important factor may contribute to the differences in the results between the two groups in addition to the disease duration itself. Therefore, we evaluated the effectiveness and safety of DBS surgery in PD patients aged older than 85 years as a separate group to consider the application of this therapy in these patients more seriously. Tables 4 and 5 show that the changes in the MDS‐UPDRS‐III motor scores and the MoCA and HAMD scores from baseline to post‐DBS surgery in these very elderly patients were similar to those in patients with longer durations of disease, as shown in Tables 2 and 3. We also did not notice an obvious increase in the preoperative main scale scores or medication usage in the subgroup aged > 85 years compared with those in the group with a disease duration ≥ 10 years, from which the subgroup was derived. In contrast, the LEDD and PDQ‐39 scores decreased, which might be due to our stricter preoperative screening of patients over 85 years old to ensure that their condition was as good as possible. Previous studies and reports specifically assessing DBS surgery for PD patients aged over 85 years are rare. We consider that these patients may be candidates for surgery if they exhibit typical PD symptoms, maintain good overall health, respond favorably to pharmacological treatment, and have normal cognitive function. This study indicated that DBS remained an effective treatment for improving the motor symptoms of these patients. Furthermore, for this subgroup of patients, cost‐effectiveness considerations are also of paramount importance. More favorable DBS cost‐effectiveness was associated with younger age and a less severe disease stage, and in China, DBS was cost‐effective for patients with advanced PD over a 15‐year time horizon [35]. Therefore, from this perspective, surgical treatment is not recommended for patients older than 85 years.

With respect to complications and adverse effects after DBS surgery, elderly patients are more likely to exhibit delirium [36, 37]. Delirium is broadly defined as the occurrence of any event of hallucinations, delusions, or disorientation to circumstances, even if it is apparently benign [38]. Previous studies have shown that age is a major risk factor for delirium following DBS surgery [36, 37, 38, 39, 40, 41, 42]. In addition to age, the disease duration [41] and severity [43] are risk factors associated with postoperative delirium. Age, a history of delirium, and disease duration were reported to be positive predictors of delirium [38]. Abboud H et al. reported that age and disease duration were noninfluential factors for postoperative confusion [43]. In particular, dysarthria, which mainly manifests as a deterioration in verbal fluency, cognitive decline, and psychosocial symptoms, occurs frequently in elderly patients after STN stimulation [26, 44]. DeLong et al. reported that increasing age (ranging from < 50 to 90 years of age) did not significantly affect 90‐day complication rates; the incidences of the two most common procedure‐related complications, hemorrhage and infection, did not significantly increase with age [13]. Their findings suggested that age alone should not be a primary exclusion criterion for determining candidacy for DBS. In this study, for elderly patients (aged ≥ 75 years), psychiatric deterioration and cognitive impairment were relatively common short‐term surgical complications. Although their incidences were not significantly different between the two groups, transient delirium and confusion had higher odds in the population with longer disease durations (40.0% vs. 21.1%). The incidence of prolonged postoperative hospitalization was not significantly different between the two groups. Nonetheless, the total incidence rate of complications in the group of patients with a long disease duration was significantly higher than that in patients with a short disease duration, indicating that DBS surgery was still considered a high‐risk procedure for these patients.

## 5 Conclusions

DBS is effective for improving motor symptoms and functions in elderly PD patients (aged ≥ 75 years) in the short and long term. Reducing the use of antiparkinsonian drugs and improving the quality of life and the ability to perform daily living activities for patients with a longer duration of disease (≥ 10 years), regardless of the onset time of disease, is difficult. Furthermore, these patients suffer relatively more short‐term complications and adverse effects. Even though patients older than 75 years are excluded from most clinical studies, we insist on carefully identifying the heterogeneity within elderly populations, and the specific age cut‐off should not be defined. Clinicians can use individual differences to guide patient selection, surgical planning, and even perioperative management. These patients should be considered for DBS surgery based on a strict examination.

## Author Contributions

Xin Wang: conceptualization, methodology, investigation, data curation, and writing – original draft; Jing Wang, Nan Li, and Hongwen Qu: formal analysis and writing – review and editing; Yuqi Wen, Bao Wang, Jian Fu, Zixuan Jing, Mingming Su, and Zhaohui Zheng: investigation and resources; Huijuan Kou, Chun Qiu: software; Xuelian Wang: project administration, supervision, and validation; Yan Qu: supervision and validation. All authors approved the final version of the manuscript.

## Conflicts of Interest

The authors declare no conflicts of interest.

## Data Availability

The data that support the findings of this study are available from the corresponding author upon reasonable request.
